# Selenium-Modified Biochar Synergistically Achieves the Safe Use of Selenium and the Inhibition of Heavy Metal Cadmium

**DOI:** 10.3390/molecules30020347

**Published:** 2025-01-16

**Authors:** Wanjing Wang, Haiyan Jiang, Zebin Tan, Luyao Yu, Jie Chen, Qingliang Xiao, Qinlei Rong, Chunhuo Zhou

**Affiliations:** 1 College of Land Resources and Environment, Jiangxi Agricultural University, Nanchang 330045, China; 2 Key Laboratory of Crop Physiology, Ecology and Genetic Breeding, Ministry of Education, Nanchang 330045, China

**Keywords:** biochar, modification, chitosan, selenium, adsorption, Cd^2+^

## Abstract

To address cadmium pollution in China’s cultivated land, chitosan, inorganic and organic selenium were used to modify rice husk charcoal for cadmium inhibition. Basic physicochemical properties of rice husk carbons were characterized (BET, FTIR, XRD, Zeta potential). Kinetic and isothermal adsorption experiments studied the adsorption of Cd^2+^ by modified biochar under different pH and dosages. A350 and C350 had pore changes, and B350 had a smoother surface. The polarity and Zeta potential of A350, B350, and C350 differed. B350 and C350’s kinetic adsorption fit the pseudo second order model, A350’s fit both the pseudo first and second order. Their isothermal adsorption fit Langmuir (B350, C350) and Freundlich (A350). Intraparticle diffusion was three-stage with single-layer chemical adsorption. The pH increase raised removal and adsorption of CK350, A350, B350, and C350. The dosage increase hiked removal but cut unit adsorption. A350 had the highest max adsorption (57.845 mg/g). All modifications enhanced Cd^2+^ adsorption, and the effect could be altered by adjusting pH and dosage.

## 1. Introduction

In recent years, a large number of human activities such as the production of nickel-cadmium batteries, pigment synthesis, metal smelting, the discharge of “three wastes” (waste gas, wastewater, and industrial residues), sewage irrigation, and the abuse of pesticides and fertilizers have led to an increase in cadmium in the soil [1]. When cadmium accumulates to a certain concentration in the environment, it will cause cadmium pollution. Some studies have shown that the area of farmland soil in China that has been polluted by heavy metals has exceeded 20 million hectares, accounting for 16.4% of the total cultivated land area, and one-third of the farmland is polluted by cadmium [2]. According to statistics, there are soil cadmium pollution problems in more than 11 provinces and 25 districts in China [3]. Soil cadmium pollution has emerged as a significant issue confronting the agricultural environment. The heavy metal cadmium is designated as a Group 1 carcinogen by the International Agency for Research on Cancer (IARC) [4]. Cadmium, a highly toxic heavy metal element, can effortlessly permeate into the human body via soil and water, thereby posing a severe risk to human health [5]. As a highly toxic and ubiquitously distributed pollutant, cadmium has a propensity to form complexes and is arduous to remediate within the soil [6]. Consequently, the remediation of cadmium-contaminated soil has invariably been a central focus of attention regarding soil health. Presently, a growing number of scholars are delving into novel approaches for rehabilitating heavy metal-contaminated soil, which entails curbing the enrichment of deleterious heavy metals in the soil while augmenting the accumulation of beneficial elements [7].

Selenium represents one of the essential trace elements required by the human body and exhibits diverse biological functions, including antioxidant, anticancer, and immune system enhancement capabilities [8]. The appropriate application of selenium fertilizer in cadmium-contaminated soil has the potential to mitigate the phytotoxicity of cadmium to plants and, to a certain extent, curtail the absorption of cadmium by plants [9,10]. This not only diminishes the risk of cadmium transfer within the food chain, but also augments rice yield, elevates the selenium content in rice, and improves rice quality [11,12]. Rice enriched with selenium demonstrates a more pronounced ability to resist cadmium pollution [11], and selenium can reduce the Cd levels in numerous crops such as rice and wheat [13,14]. Studies have revealed that under Cd stress, low selenium treatment can increase the number of pods per plant by 20.92% and significantly decrease the cadmium content in different organs of rapeseed by 4.74–26.89% [15]. Xu et al. discovered that the application of selenium can impede the accumulation and transfer of Cd in rice seedlings, thereby reducing the toxic impacts of Cd on the growth and development of rice [16]. However, selenium represents a double-edged sword with respect to soil and human health.

Selenium is an element that occurs naturally in the Earth’s crust, and high concentrations of selenium are toxic. In agricultural production, selenium can amass in the soil via evaporation and infiltrate into agricultural drainage systems. Selenium that enters surface water can be further concentrated through evaporation, generating toxins for organisms in the water, such as fish. Excessive selenium intake by humans can precipitate acute or chronic selenium poisoning [17]. For instance, selenium poisoning incidents have transpired in Kesterson, California, Punjab, India, and Enshi, Hubei, China [18]. Soil selenium contamination escalates the selenium levels in the edible portions of crops, and excessive selenium entering the human body can induce poisoning, leading to hair and nail loss, neurological diseases, etc. [19,20]. Soil selenium pollution affects the growth and development of plants, primarily manifested as low concentrations of selenium promoting plant growth and development, while high concentrations of selenium exert inhibitory effects [21]. Consequently, excessive selenium can suppress plant growth and attenuate plant resistance and antioxidant activity, endangering the growth and development of plants [22].

Biochar, as a material for passivation, has been demonstrated to effectively reduce the availability of cadmium in soil [17]. The application of biochar materials can modify the form of heavy metals in the soil, diminishing their migration rate and bioavailability in heavy metal-contaminated soil [23]. Research by Park et al. [24] indicated that biochar can reduce the solubility of cadmium in soil and enhance the nutrient absorption efficiency of crops, alleviating the bioavailability of heavy metals to plants. The addition of biochar conspicuously reduced the accumulation of Cd in the underground parts of corn, with a reduction of 51.2% [25]. The capacity of biochar to remediate cadmium-contaminated soil is correlated with its own physicochemical properties, and it is frequently modified with acids, bases, oxidants, and metal ions [26], further augmenting its potential for soil remediation. Zhang et al. [27] prepared biochar from corn straw and modified it with H_2_O_2_. The modified biochar exhibited an increased specific surface area, acidity, and polarity, accompanied by a greater number of oxygen-containing functional groups on the surface. Wang et al. [28] modified biochar with different concentrations of alkali, and the rice husk biochar treated with medium concentration alkali was optimal for stabilizing heavy metals Cd and Pb in the soil. Additionally, activated biochar prepared by high-temperature pyrolysis with supplementary gases such as CO_2_ or steam can expand its pore size and enhance its specific surface area [29]. By incorporating inorganic substances such as metal salts, halogens, or other heteroatoms, the types, quantities, and active sites of functional groups on the surface of biochar can be altered, enhancing its performance and specificity. Liu et al. [30] modified lignin porous biochar with KMnO_4_, MnSO_4_, and MnO_2_, enhancing the adsorption capacity and efficiency of the modified biochar for organic dyes. Lian et al. [31] synthesized N-doped biochar in NH_3_, substantially increasing the micropore rate and specific surface area of the biochar and forming Lewis bases on the surface of the biochar. Lu et al. [32] treated eucalyptus waste with acid and, in comparison with untreated biochar, the modified biochar possessed a larger surface area, thereby improving the adsorption performance for heavy metals.

Previous studies have combined biochar with foliar selenium fertilizer for research purposes. Zhao et al. ascertained that the combined application of foliar selenium and biochar can effectively promote the enrichment of selenium in peanuts, suppress Cd toxicity, and remarkably increase the biomass of peanuts by 73.44–132.41% [33]. There is relatively scant research on the selenium modification of biochar. Selenium is an element in the fourth period, Group VIA of the periodic table, and it exhibits an antagonistic effect on the accumulation of heavy metals in plants, which can mitigate their inhibitory effects on plant growth [34]. Owing to its similar chemical properties to sulfur, selenium can chelate with heavy metals to form selenium-heavy metal complexes, reducing their toxicity to plants [35]. Therefore, this study employed inorganic selenium, organic selenium, and chitosan-made chitosan selenium as an intermediate medium to modify rice husk biochar with the aim of enhancing its cadmium ion removal efficiency. This study explores the alterations in cadmium adsorption.

## 2. Results

### 2.1. Characterization of Rice Husk Biocharn

#### 2.1.1. SEM

**Figure 1:**
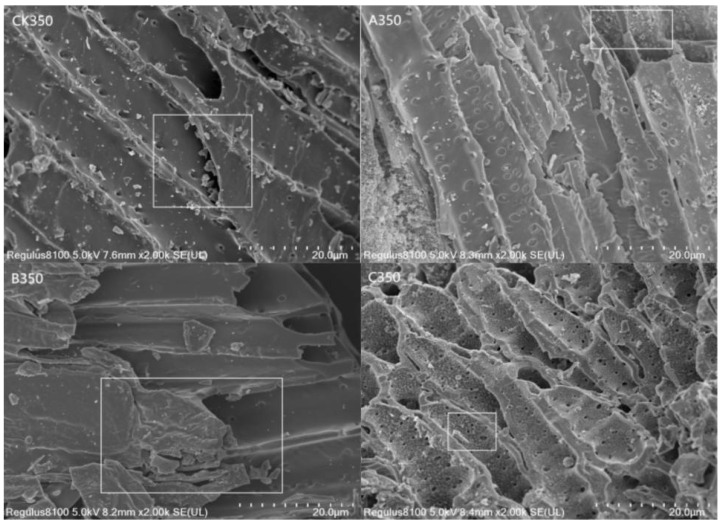
Scanning electron microscopy of rice husk biochar and selenium-modified rice husk biochar (White boxes: comparison of the surface morphology of biochar before and after modification).

As can be seen from Figure 1: the surface of the CK350 prepared in this study was relatively rough with noticeable granular material. A350 and C350 had uniform particle sizes, clear stratification, and significantly increased pores, providing a fluffy structure that offered a richer ideal site for effective adsorption. The surface of B350 became smoother, with most of the pores filled.

#### 2.1.2. Elemental Composition

The elemental content of rice husk biochar after different modification treatments is presented in Table 1. As can be seen from Table 1, compared to the Se content of CK350, A350 increased by 4.5%, B350 by 3.56%, and C350 by 2.67%. This indicates that the Se element was successfully incorporated onto the rice husk biochar. At a pyrolysis temperature of 350 °C, the molar ratios of O/C and (O+N)/C of the rice husk biochar indicate its hydrophilicity and polarity.

#### 2.1.3. Fourier Transform Infrared Spectroscopy (FTIR)

**Figure 2:**
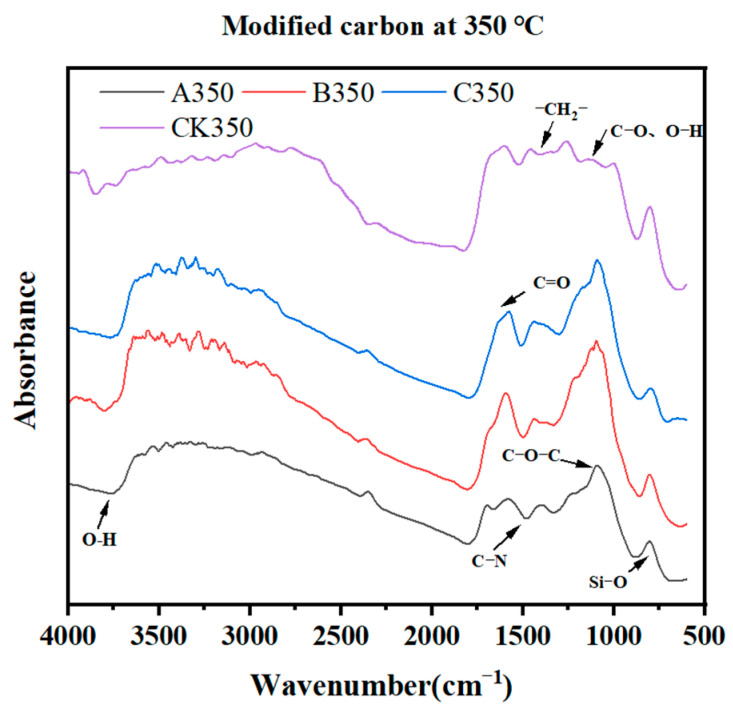
Fourier transform infrared spectroscopy of rice husk biochar and selenium-modified rice husk biochar.

The results of Fourier transform infrared spectroscopy (FTIR) analysis of rice husk biochar after different modification treatments are shown in Figure 2. Most of the absorption peaks of the four biocarbons appeared at the same position. Compared with CK350, the -OH stretching vibration peak was exhibited near 3700 cm^−1^ [36], indicating that the -OH group in the rice husk biochar had a chemical reaction with the modified selenium-containing substance. The peak near 1700 cm^−1^ belonged to the C=O stretching vibration of carboxyl, ketone, aldehyde, and ester groups [37], which was enhanced after modification, indicating that the modified substance contained richer oxygen-containing functional groups. A350, B350, and C350 exhibited vibration caused by ether bond O-C-O at 1150 cm^−1^ [38], which was enhanced after modification, indicating that the modified selenium has been successfully grafted to rice. On the shell biochar, four kinds of biochar showed an Si-O stretching vibration peak at 801 cm^−1^.

**Figure 3:**
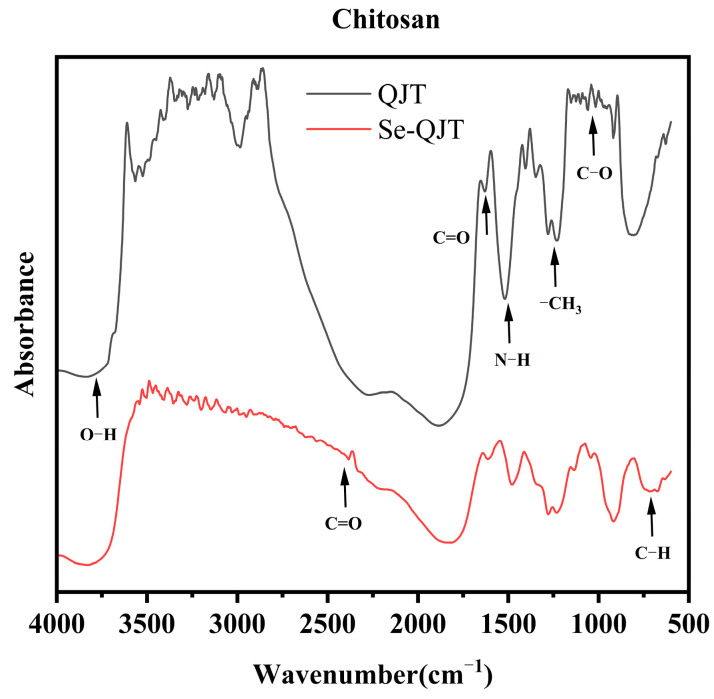
Fourier transform infrared spectroscopy of chitosan and selenium-chitosan.

The FTIR analysis results for chitosan and selenium-chitosan are depicted in Figure 3. The band near 1500 cm^−1^ is assigned to the bending vibration peak of the amine group. The absorption peak near 1030 cm^−1^ is the C-O stretching vibration absorption peak. The characteristic absorption at 1700 cm^−1^ is the C=O stretching vibration peak. The bending vibration peak of the amine group at 1500 cm^−1^ decreases. The -H stretching vibration appears around 2800 cm^−1^. The C-O stretching vibration near 1200 cm^−1^ decreases. The new absorption peak at 710 cm^−1^ is produced by the bending vibration of C-H in the aromatic ring. In summary, these observations indicate the successful incorporation of inorganic selenium into QJT.

#### 2.1.4. X-Ray Diffraction (XRD) Analysis

**Figure 4:**
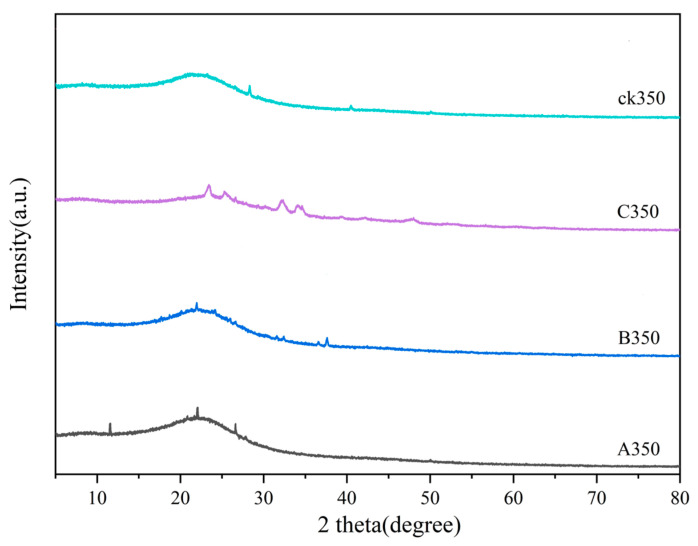
X-ray diffraction of rice husk biochar and selenium-modified rice husk biochar.

As shown in Figure 4, the characteristic peak of the SiO_2_ crystal plane appears at 2θ = 26° for both rice husk biochar and selenium-modified rice husk biochar, indicating the presence of a small amount of impurity SiO_2_, which is an amorphous characteristic peak [39]. For C350, a characteristic peak of ammonia was observed at 2θ of 34.02°, and a selenium elemental characteristic peak appeared at 2θ of 24.01°, indicating that selenomethionine was successfully grafted onto the rice husk biochar. A350 exhibited a diffraction absorption peak of chitosan at 2θ of 11.05° [40,41], indicating that selenium-chitosan was successfully loaded, and a characteristic peak of Na_2_SeO_3_ was observed at 2θ of 24.08°. B350 showed a characteristic peak of Na_2_SeO_3_ at 2θ of 22.03° [42,43]. The appearance of a hump peak at the same position for CK350, A350, and B350 suggests that the internal crystal structure of these modified biochars did not changed after modification.

#### 2.1.5. Zeta Potential

**Figure 5:**
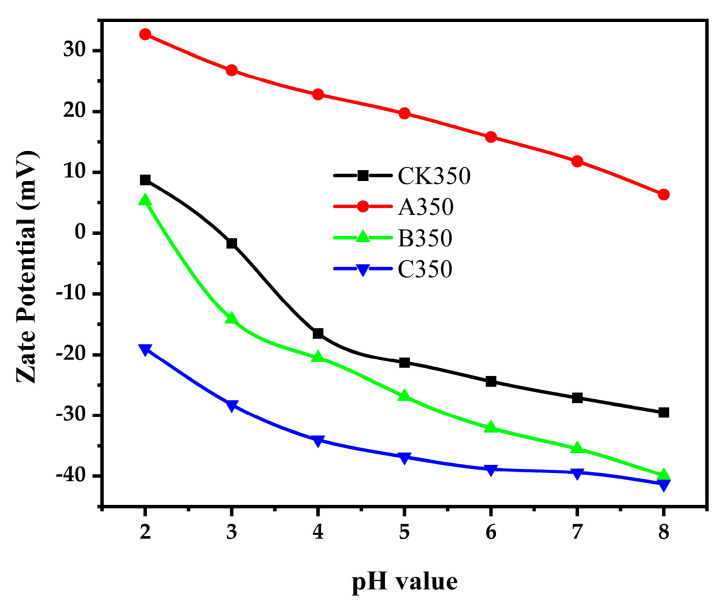
Zeta potential of rice husk biochar and selenium-modified rice husk biochar.

Zeta potential is used to characterize the surface charge parameters of charged particles, to analyze the mutual repulsive and attractive forces between charged particles, and to reveal the macroscopic phenomena of interactions between particles [44]. The polarity of biochar on its surface is closely related to the functional groups it contains. For example, the presence of polar functional groups such as hydroxyl groups and carboxyl groups will enhance the polarity of biochar. The dissociation or adsorption behaviors of these functional groups will make the surface of biochar charged, which will further affect its Zeta potential [45]. When the pH value of the solution increases, the acidic functional groups on the surface of biochar may further dissociate, resulting in an increase in surface charge, a decrease in Zeta potential, and an enhancement of polarity. However, when the ionic strength increases, the ions in the solution will have a shielding effect on the charge on the surface of biochar, leading to an increase in Zeta potential and a relative weakening of the polarity [46]. As shown in Figure 5, the Zeta potential of the four materials decreases with the increase in pH, indicating that the adsorption capacity of rice husk biochar and its modified forms for Cd^2+^ increases with the increase in pH. When the Zeta potential is zero, the pHpzc of CK350 is 2.92, and that of B350 is 2.46, while C350 maintains a negative charge at any pH. The amount of charge is related to the number of functional groups such as hydroxyl and carboxyl groups on the biochar; the more such functional groups, the higher the negative charge, and the greater the electrostatic repulsion between biochar particles, which is more favorable for the adsorption of metal cations [38]. After modification, compared with CK350, the polarity of A350 increased, while the polarities of B350 and C350 decreased. With a negative Zeta potential, C350 undergoes deprotonation, carries a negative charge, and has an electrostatic attraction with Cd^2+^ [39]. A350 maintains a positive charge at any pH, with its protonated surface carrying a positive charge, and has electrostatic repulsion with Cd^2+^, suggesting that complexation reaction may be the main reason for the adsorption of Cd^2+^ by A350.

### 2.2. Adsorption Characteristics of Biochar

#### 2.2.1. Adsorption Kinetics

**Figure 6:**
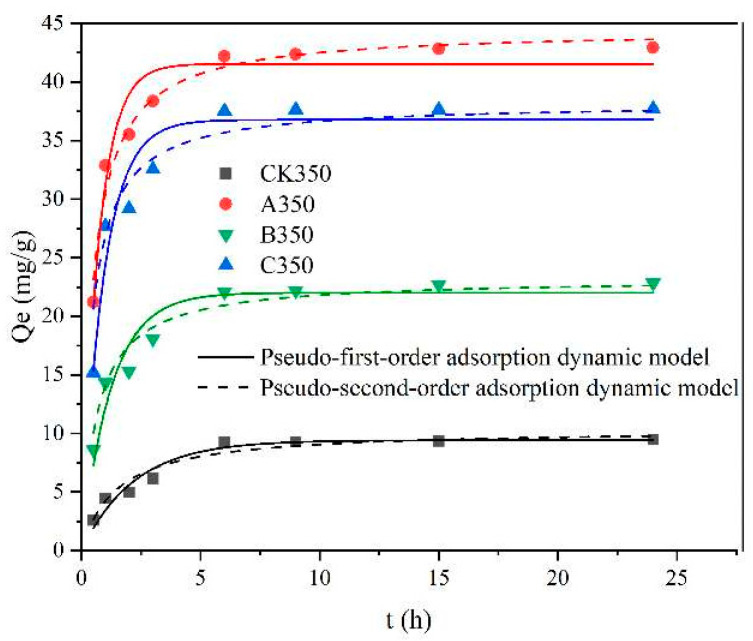
Adsorption kinetic model fitting curves of rice husk biochar and selenium-modified rice husk biochar.

Figure 6 and Table 2 show the fitting results and related parameters of the pseudo-first order and pseudo-second order kinetic models for the adsorption of heavy metal Cd^2+^ by CK350, A350, B350, and C350. The biochars exhibited a rapid adsorption rate for Cd^2+^ in the first 12 h, then leveled off, and eventually reached equilibrium. In Table 2, the correlation coefficient R^2^ of the pseudo-first order kinetic model for CK350, B350, and C350 is less than 0.9, indicating average linear correlation. However, the correlation coefficient R^2^ for the pseudo-second order kinetic model for CK350, A350, B350, and C350 is greater than 0.9, indicating good linear correlation [47]. This suggests that the adsorption process of Cd^2+^ by CK350, B350, and C350 is more consistent with the pseudo-second order kinetic model, and the theoretical adsorption values are also closest to the actual values, with equilibrium adsorption amounts of 10.379 mg/g, 24.233 mg/g, and 36.211 mg/g. The pseudo-second order kinetic model assumes that the adsorption rate is controlled by chemical adsorption mechanisms; hence, the rate constant (K_2_) is determined by the chemical adsorption rate of heavy metals on biochar. The larger the value of K_2_, the higher the adsorption rate and the shorter the time to reach equilibrium [48]. The K_2_ values for the four types of biochars are in the order A350 > C350 > B350 > CK350, indicating that A350 reaches adsorption equilibrium more quickly. Notably, the correlation coefficient R^2^ for both models for A350 is greater than 0.9.

**Figure 7:**
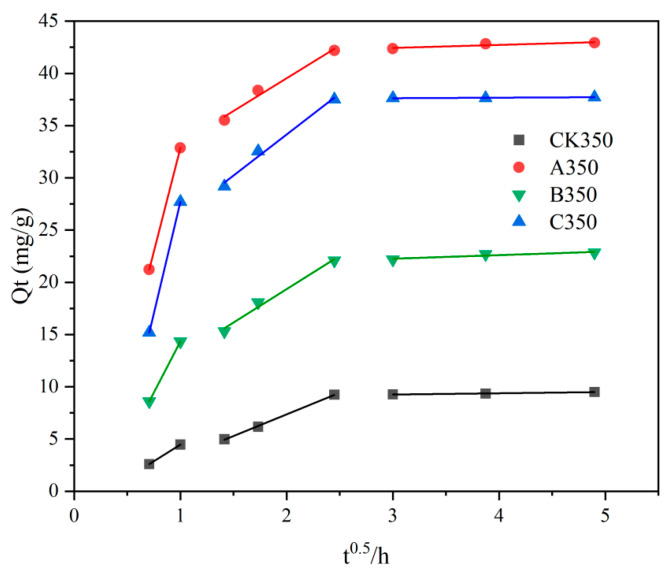
Fitting chart of the intraparticle diffusion model for rice husk biochar and selenium-modified rice husk biochar.

The fitting results of the intraparticle diffusion model are shown in Figure 7 and Table 3. The Cd^2+^ adsorption process can be clearly divided into three stages as follows: rapid removal stage (0–1 h), slow removal stage (2–6 h), and near-equilibrium stage (9–24 h) [49]. In the third stage, the adsorption rates of the four types of biochars from high to low were A350 > C350 > B350 > CK350. As the concentration difference between the solid–liquid phases further decreases, the effective adsorption sites gradually become saturated, and the entire system exhibits a dynamic equilibrium or near-equilibrium state [50].

#### 2.2.2. Adsorption Isotherms

**Figure 8:**
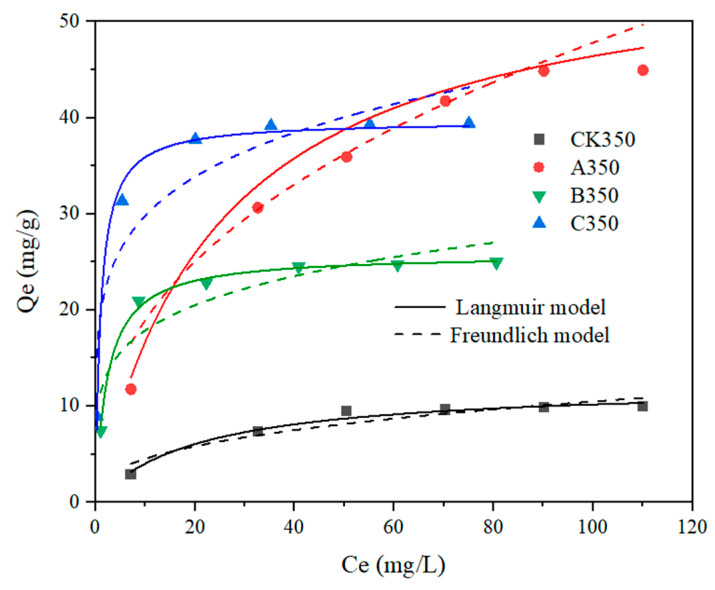
Fitting curves of the isothermal adsorption models for rice husk biochar and selenium-modified rice husk biochar.

The Langmuir and Freundlich isothermal adsorption models were used to fit the adsorption process of Cd^2+^ by rice husk biochar and selenium-modified rice husk biochar, with the results presented in Table 4 and Figure 8. As the initial concentration of Cd^2+^ increases, the adsorption amount by each type of biochar also increases, approaching equilibrium as the active sites become saturated.

Table 4 indicates that the R^2^ values for the Freundlich model fitting equations for CK350, B350, and C350 are larger than those for the Langmuir model fitting equations, with maximum adsorption capacities reaching 12.256 mg/g, 39.666 mg/g, and 25.799 mg/g, respectively. This suggests that the adsorption process of heavy metal Cd^2+^ by CK350, B350, and C350 is more consistent with the Freundlich model. It indicates that the adsorption process of heavy metal Cd^2+^ by CK350, B350, and C350 tends towards monolayer adsorption, with Cd^2+^ being adsorbed more extensively on the surface of the biochar. For A350, the R^2^ value for the Langmuir model fitting equation is larger than that for the Freundlich model fitting equation, indicating that the adsorption process of heavy metal Cd^2+^ by A350 is more consistent with the Langmuir model.

#### 2.2.3. The Impact of the Initial Solution pH on the Adsorption of Cd^2+^ by Selenium-Modified Rice Husk Biochar

**Figure 9:**
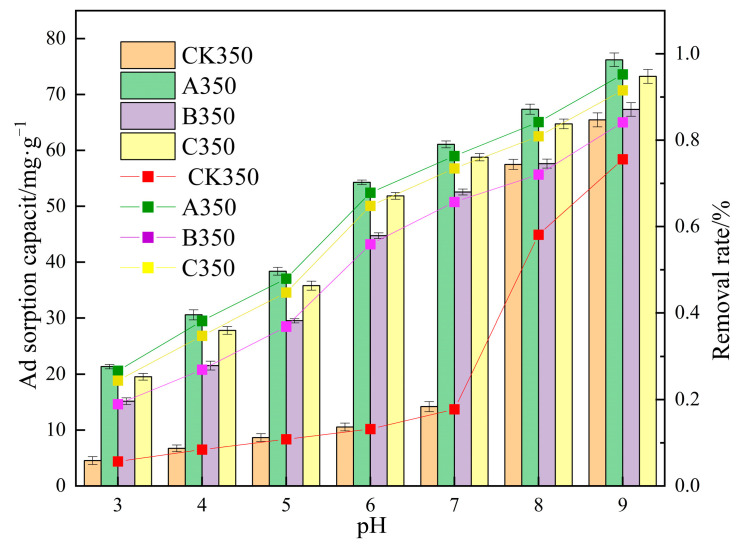
The effect of different solution pH conditions on the adsorption of Cd2+ by selenium-rice husk biochar.

The results of the adsorption amount and removal rate of Cd^2+^ by various rice husk biochars under different pH conditions are shown in Figure 9. The removal rate and adsorption amount of Cd^2+^ by CK350, A350, B350, and C350 increase with the rise of solution pH. When the pH increases from 3 to 7, the removal rate of Cd^2+^ improves, with the removal rates of CK350, A350, B350, and C350 increasing from 5.69%, 26.70%, 24.43%, and 18.95% to 17.75%, 76.34%, 73.47%, and 65.69%, respectively, and the adsorption amounts increasing from 4.54, 21.36, 19.54, 15.16 mg/g to 14.20, 61.07, 58.77, 52.55 mg/g, respectively. Under acidic and neutral conditions, the removal rate and adsorption amount of A350, B350, and C350 are always higher than those of CK350. When the pH increases from 8 to 9, the removal rates of CK350, A350, B350, and C350 increase from 58.11%, 84.20%, 80.91%, and 72.04% to 75.58%, 95.27%, 91.55%, and 84.15%, respectively, and the adsorption amounts increase from 46.485, 67.361, 64.73, 57.63 mg/g to 60.467, 76.21, 73.23, 67.32 mg/g, respectively. Under slightly alkaline conditions (pH = 8), the removal rate and adsorption amount of A350, B350, and C350 are all higher than those of CK350. As the pH continues to rise (pH = 9), the removal rate and adsorption amount of CK350, A350, B350, and C350 tend to approach each other.

#### 2.2.4. The Effect of Dosage on the Adsorption of Cd^2+^ by Selenium-Modified Rice Husk Biochar

**Figure 10:**
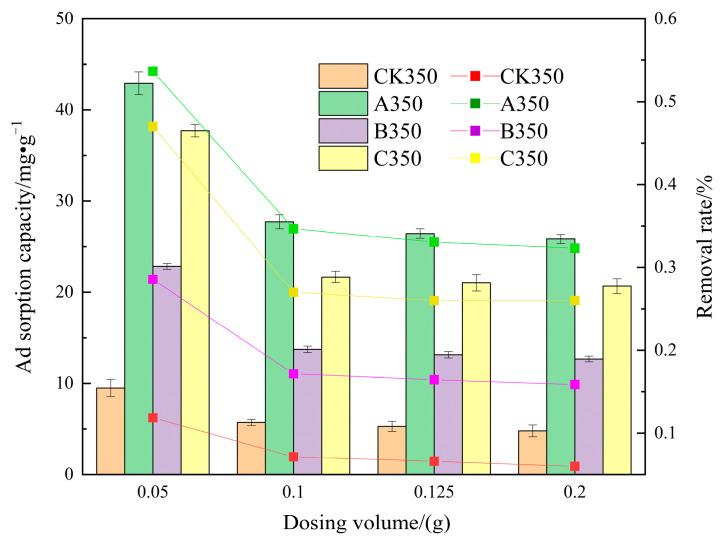
The effect of different dosages on the adsorption of Cd^2+^ by selenium-modified rice husk biochar.

The effect of the dosage of CK350, A350, B350, and C350 on the adsorption of Cd^2+^ is shown in Figure 10. When the dosage of CK350, A350, B350, and C350 is increased from 0.05 g to 0.1 g, the removal rates decrease from 11.87%, 53.66%, 47.16%, and 28.56% to 7.15%, 34.68%, 27.10%, and 17.17%, respectively, and the adsorption amounts of Cd^2+^ decrease from 9.49, 42.92, 37.72, 22.84 mg/g to 5.72, 27.74, 21.68, 13.74 mg/g, respectively. When the dosage is increased from 0.1 g to 0.2 g, the increase in the removal rate of the four types of biochar is relatively small and tends to be balanced, but the adsorption amount per unit decreases. Under the same dosage conditions, the removal rate and adsorption amount of A350, B350, and C350 are all better than those of CK350.

## 3. Discussion

To investigate the adsorption mechanism of modified biochar towards cadmium ions, the adsorption characteristic outcomes have demonstrated that the adsorption process of Cd^2+^ by the four varieties of biochar is not merely restricted by intraparticle diffusion but is also affected by other comprehensive elements that impact the entire adsorption procedure [51]. The findings from the intraparticle diffusion model suggest that A350, B350, and C350 possess relatively rapid intraparticle diffusion rates [52], implying that the adsorption of Cd^2+^ by A350, B350, and C350 is a multi-step and multi-stage process [53]. The adsorption process of heavy metal Cd^2+^ by these three types of biochar might encompass ion exchange, surface complexation, and surface precipitation, and is predominantly governed by chemical adsorption mechanisms [54].

In this study, the adsorption performance of the modified rice husk charcoal for Cd^2+^ is shown as follows: A350 > C350 > B350 > CK350. The action mechanisms of the three types of modified biochar in adsorbing Cd^2+^ are different. Firstly, the surfaces of A350 and C350 become rough with dense granular protrusions, which makes the specific surface area of A350 and C350 increase compared with that of CK350. With a larger specific surface area, the pore structure is more developed and more complex. Secondly, the amplitude of the absorption peak caused by the surface -OH groups of A350, B350, and C350 increases compared with that before modification, indicating that the surface -OH groups of the selenium-modified biochar increase. Previous studies have shown that the -OH on the surface of biochar has a chemical complexation effect with Cd^2+^, which is an important mechanism for biochar to adsorb Cd^2+^. It can be seen that after selenium modification, both the physical and chemical adsorption capacities of biochar for Cd^2+^ are enhanced. The maximum adsorption amount of A350 for Cd^2+^ is 4.72 times that of the unmodified biochar. The aromaticity of A350 is enhanced, while its hydrophilicity and polarity are weakened, which is beneficial to improving the surface adsorption capacity of biochar and increasing the adsorption sites on the surface of A350. The Zeta potential shows that the Zeta potential of A350 is positive under any pH, and the complexation reaction enhances its adsorption capacity for Cd^2+^. The adsorption kinetic results indicate that the adsorption of Cd^2+^ by A350 is controlled by the physical diffusion mechanism and the chemical adsorption mechanism. Secondly, the adsorption performance of C350 for Cd^2+^ is slightly lower than that of A350. The Zeta potential shows that the electrostatic repulsion among the particles of C350 is large, which is beneficial to adsorbing metal cations. The fitting of the Langmuir model indicates that the adsorption process of Cd^2+^ in the solution by C350 is mainly monolayer adsorption. In this study, the maximum adsorption amount of B350 for Cd^2+^ was 3.23 times that of the unmodified biochar. In addition, in this study, the adsorption performance of B350 for Cd^2+^ was relatively weak. This may be due to the fact that after modification, the surface morphology of the biochar becomes smooth, and both the specific surface area and the pore volume decrease. However, compared with the control group, its surface -OH groups also increase, and the adsorption sites for Cd^2+^ also increase accordingly.

With the elevation in pH, the adsorption capacity of biochar for Cd^2+^ also augments. At an acidic pH, the increment in the removal rate is relatively modest. This could be attributed to the fact that at lower solution pH, the adsorption sites on the surface of the charcoal particles are occupied by a substantial quantity of H^+^ ions, inducing electrostatic repulsion between Cd^2+^ and the adsorption sites, as H+ and Cd^2+^ compete for adsorption sites [55,56]. As the pH rises, the competition between H^+^ and Cd^2+^ diminishes, facilitating the binding of Cd^2+^ with negatively charged binding sites on the surface, and the removal efficiency of Cd^2+^ by each modified rice husk biochar persists in increasing. Under weakly alkaline conditions, the functional groups on A350, B350, and C350 are deprotonated, augmenting the negative charge on the surface of the charcoal, reducing the electrostatic repulsion and competition forces, and liberating more Cd^2+^ adsorption sites from the negatively charged functional groups [57]. Consequently, the removal efficiency of Cd^2+^ by A350, B350, and C350 is enhanced, and the adsorption capacity is amplified. Under strongly alkaline conditions (pH = 9), the solution becomes alkaline, and the number of OH^−^ ions progressively escalates, leading to the precipitation of Cd(OH)_2_ [58].

The removal rate of Cd^2+^ from the solution by the four types of biochar escalates with the increase in the added amount, which might be because the dosage of CK350, A350, B350, and C350 is directly proportional to the number of active sites; the greater the adsorbent dosage, the more active sites are available [59], thereby augmenting the removal rate of Cd^2+^. As the amount of biochar added continues to increase, the number of adsorption sites on the surface of the biochar that have not attained saturation adsorption persists in increasing, resulting in a reduction in the Cd^2+^ adsorption amount per unit amount of biochar [60].

When comparing CK350, B350, and C350, A350 exhibited the highest adsorption capacity and the most rapid adsorption rate for Cd^2+^. The fitting results of the two adsorption kinetic models for A350 possess a correlation coefficient R^2^>0.9, signifying that the adsorption process of A350 for heavy metal Cd^2+^ is more in line with the Langmuir model. The Langmuir model is derived from the monolayer physical adsorption of adsorbates on open surfaces, whereas the Freundlich model is appropriate for simulating multilayer adsorption equilibrium on non-uniform surfaces of adsorbents [61], suggesting that A350 might possess both physical and chemical adsorption, with a more expeditious adsorption equilibrium for Cd^2+^ and a non-uniform multilayer adsorption process. It is plausible that after modification with chitosan selenide, the polarity of A350 is diminished and the number of oxygen-containing functional groups is decreased, rendering it more facile to form stable aromatic structures, and the hydrophilicity is attenuated, which is congruent with the Zeta potential results. This indicates that the introduction of chitosan as an intermediate not only successfully embeds sodium selenite but also remarkably augments the adsorption capacity of the modified biochar for Cd^2+^.

In comparison with existing research, the adsorption capacities of biochar modified by different methods for Cd^2+^ are different. For example, the maximum adsorption amount of rice husk charcoal pyrolyzed at 500 °C for Cd^2+^ reaches 4.23 mg/g [62]. The maximum adsorption amount of chitosan-modified biochar prepared from corn stalks for Cd^2+^ reaches 28.64 mg/g [41]. The maximum adsorption amount of iron-modified biochar prepared by the impregnation method for Cd^2+^ is 49.78 mg/g [63]. In this study, the selenium-modified biochar A350 has achieved a high adsorption capacity of 57.845 mg/g, which is significantly better than those of unmodified or other conventionally modified biochar, fully demonstrating the significant advantages of selenium modification in improving the adsorption performance of biochar.

In the actual environment, the conditions such as the pH and ionic strength of soil and water are complex and variable. Due to its unique surface properties and adsorption mechanism, the selenium-modified biochar can exhibit good adsorption performance under different pH conditions, and the changing trend of its adsorption capacity with the change of pH also reflects certain regularity and adaptability. In contrast, the adsorption performance of some traditionally modified biochar may decline significantly outside a specific pH range, indicating that the selenium-modified biochar has better advantages in dealing with complex environmental conditions and is more capable of meeting the needs of actual pollution remediation scenarios.

## 4. Materials and Methods

### 4.1. Reagents and Principles

This experiment selected rice husks as the raw material for preparing biochar. The main chemical reagents used for the modification of rice husk biochar were as follows: chitosan (Sinopharm Chemical Reagent Co., Ltd., Shanghai, China), anhydrous ethanol (Xilong Scientific Co., Ltd., Shantou, China), sodium selenite (Xilong Scientific Co., Ltd., Shantou, China), selenomethionine (Xilong Scientific Co., Ltd., Shantou, China), glutaraldehyde (Xilong Scientific Co., Ltd., Shantou, China), and sodium sulfide (Xilong Scientific Co., Ltd., Shantou, China). The main chemical reagents used in the adsorption tests were as follows: cadmium nitrate tetrahydrate (Xilong Scientific Co., Ltd., Shantou, China), sodium hydroxide (Xilong Scientific Co., Ltd., Shantou, China), hydrochloric acid (Xilong Scientific Co., Ltd., Shantou, China), and sodium nitrate (Xilong Scientific Co., Ltd., Shantou, China), all of which are of analytical reagent (AR) grade.

### 4.2. Preparation and Modification of Rice Husk Biochar

Preparation of rice husk biochar: The particulate impurities in rice husks were removed by washing with deionized water. Then, the rice husks were dried in a drying oven (DHG-9101-1SA, Shanghai Hongdu Electronic Technology Co., Ltd., Shanghai, China) at a constant temperature of 60 °C. The rice husks were filled and compacted in an aluminum foil paper box and then placed in a muffle furnace (SX2-4-10, Shanghai Super Information Technology Co., Ltd., Shanghai, China), with the pyrolysis temperature set at 350 °C. After reaching the initially set temperature, the temperature was kept constant for 2 h. After natural cooling, the product was ground with a ceramic mortar and then passed through a 100-mesh plastic sieve to obtain the final product.

Preparation of Selenium-Chitosan-Modified Rice Husk Charcoal: In total, 8 g (accurate to 0.0001 g) of chitosan was accurately weighed and dissolved it in 800 mL of 1% acetic acid (HAc) solution. Then, it was placed on a magnetic stirrer (MYP11-2A, Shanghai Meiyingpu Instrument and Meter Manufacturing Co., Ltd., Shanghai, China) and stirred evenly. Slowly, a certain amount of sodium selenite was added and stirring continued evenly on the magnetic stirrer. After standing still, it was placed in a centrifuge (TG16-WS, Hunan Xiangyi Laboratory Instrument Development Co., Ltd., Changsha, China) and centrifuged at 3000 r·min for 10 min. Taking the supernatant, 95% ethanol was added at a ratio of 1:1 for sufficient precipitation. After standing still again, it was placed in the centrifuge and centrifuged at 3000 r/min for 10 min. The washing was repeated with absolute ethanol twice, dried at a low temperature, and then ground with a ceramic mortar, and passed through a 100-mesh plastic nylon sieve to obtain selenium-chitosan.

In total, 4 g (accurate to 0.0001 g) of selenium-chitosan was dissolved in 800 mL of 1% HAc solution. It was placed on a magnetic stirrer and stirred evenly. Then, 4 g (accurate to 0.0001 g) of rice husk charcoal as added. After stirring, 6 mL of glutaraldehyde was added and it was placed on a magnetic stirring water bath (HH-2J, Changzhou Langyue Instrument Manufacturing Co., Ltd., Changzhou, China) at 50 °C and stirred evenly. It was washed with deionized water while performing suction filtration. It was then dried at a low temperature, ground with a ceramic mortar, and passed through a 100-mesh plastic nylon sieve to obtain selenium-chitosan-modified rice husk charcoal, which was used for subsequent characterization and adsorption experiments.

Preparation of Inorganic Selenium-Modified Rice Husk Charcoal: Accurately, 2 g (accurate to 0.0001 g) of sodium selenite was weighed and dissolved in 800 mL of deionized water. It was placed on a magnetic stirrer and stirred evenly. Then, 6 g (accurate to 0.0001 g) of rice husk charcoal was added, and it was placed on a magnetic stirring water bath at 60 °C and stirred evenly. After standing still, the supernatant was separated. It was washed with deionized water while performing suction filtration. It was then dried at a low temperature, ground with a ceramic mortar, and passed through a 100-mesh plastic nylon sieve. This was used for subsequent characterization and adsorption experiments.

Preparation of Organic Selenium-Modified Rice Husk Charcoal: The rice husk charcoal (in grams), N,N-dimethylformamide (DMF, as the reaction medium in milliliters), selenomethionine (in grams), sodium sulfide nonahydrate (in grams), and absolute ethanol (in milliliters) were prepared in a ratio of 2:5:10:12:50. Sequentially, the rice husk charcoal, DMF, and selenomethionine were added into a beaker according to the above ratio, and 30 g (accurate to 0.01 g) of sodium bisulfate was added as a catalyst. This was reacted at 120 °C for 4 h. After the reaction was completed, it was cooled down to room temperature. Then, sodium sulfide nonahydrate and absolute ethanol were added according to the above ratio. This was reacted for 2 h under magnetic stirring, and then vacuum filtration was performed with a suction filtration pump (using a 1.0 μm water-based filter membrane). It was then washed with deionized water while performing suction filtration. It was then ground with a ceramic mortar and passed through a 100-mesh plastic nylon sieve. This was for subsequent characterization and adsorption experiments.

### 4.3. Characterization of Modified Biochar

Scanning electron microscopy (SEM) was performed using a cold field emission scanning electron microscope (Regulus8100, Hitachi High-Tech Corporation, Tokyo, Japan) to analyze the samples. Fourier transform infrared spectroscopy (FTIR, NicoletiS iS50, Theemo Fisher Scientific Co, Agawam, MA, USA) potassium bromide pellet method was used to conduct structural analysis of the characteristic functional groups of the samples within the wavenumber range of 400–4000 cm^−1^. X-ray diffraction (XRD, SmartLab 3 KW, Rigaku, The Woodlands, TX, USA) was used to determine the crystal diffraction pattern of the samples. A Zeta potential analyzer (Zetasizer Nano ZS90, Malvern Panalytical, Malvern, UK) was used to measure the zero point charge of the samples at different pH levels.

### 4.4. Adsorption Tests

#### 4.4.1. Adsorption Kinetics Test

A Cd**^2+^** solution was prepared with a concentration of 80 mg/L using Cd(NO_3_)_2_·4H_2_O (with a background solution of 0.01 mol/L NaNO_3_). In total, 25 mL of the above solution and 0.05 g (accurate to 0.0001 g) of the above adsorption material were added into a 50 mL centrifuge tube. Then, the centrifuge tube was placed in a constant temperature water bath shaker (SHA-C, Changzhou Runhua Electric Appliance Co., Ltd., Changzhou, China) and constant temperature oscillation at 25 °C and 220 rpm/min was conducted. This was sampled at 30, 60, 120, 180, 360, 720, 900, and 1440 min, respectively (with 3 replicates set for each time point). The samples were placed in a centrifuge and centrifuged at 4000 r/min for 5 min. A certain amount of the supernatant was taken and filtered under reduced pressure using a suction filtration pump (GM-0.2, Tianjin Jinteng, Tianjin, China) through a 0.45 μm water-based filter membrane. An inductively coupled plasma mass spectrometer (iCAP-Q MS, Thermo Fisher Scientific China Co., Ltd. Waltam, MA, USA) was used to determine the concentration of Cd**^2+^** in each solution sample, and then the adsorption amount of Cd**^2+^** by each biochar was calculated and the kinetic model was fitted.

#### 4.4.2. Isothermal Adsorption Test

Cd^2+^ solutions were prepared with initial concentrations of 40, 80, 100, and 120 mg/L, respectively. In total 25 mL of the Cd^2+^ solutions was taken with a series of concentration gradients and 0.05 g (accurate to 0.0001 g) of the above-mentioned adsorption materials and were added 50-mL centrifuge tubes (with 3 parallel samples set for each concentration). They were placed in a constant-temperature water-bath shaker and constant-temperature oscillation at 25 °C and 220 rpm/min was conducted. After 24 h, the centrifuge tubes were taken out and placed in a centrifuge at 4000 r/min for 5 min. A certain amount of the supernatant was taken and filtered under reduced pressure using a suction-filtration pump through a 0.45-μm water-based filter membrane. Inductively-coupled plasma mass spectrometry was used to determine the Cd^2+^ concentration of each solution sample. Then, the adsorption amount of Cd^2+^ was calculated by each biochar and fit the isothermal–adsorption model.

#### 4.4.3. The Influence of the Initial Solution pH Value on the Adsorption Experiment

In total, 0.05 g (accurate to 0.0001 g) of the adsorption material was weighed and added into 25-mL Cd^2+^ solution with an initial concentration of 80 mg/L, whose initial pH values were adjusted to 3, 4, 5, 6, 7, 8, and 9, respectively, using 0.1 mol·L^−1^ NaOH and 0.1 mol·L^−1^ HCl. These solutions were placed in centrifuge tubes (with 3 parallel samples for each pH value). The were oscillated at a constant temperature of 25 °C and a speed of 220 rpm/min in a constant-temperature water-bath shaker. After 24 h, the centrifuge tubes were taken out and placed in a centrifuge at a speed of 4000 r/min for 5 min. A certain amount of the supernatant was taken and filtered under reduced pressure using a suction-filtration pump through a 0.45-μm water-based filter membrane. Inductively-coupled plasma mass spectrometry was used to determine the Cd^2+^ concentration of each solution sample. Then, the adsorption amount and removal rate of Cd^2+^ by each biochar at different pH values were calculated.

#### 4.4.4. Adsorption Test at Different Addition Amounts

At 25 °C, a Cd^2+^ solution with a mass concentration of 80 mg/L was prepared. Accurately, 25 mL of the solution was measured and added into 50-mL centrifuge tubes, respectively. Then, accurately 0.05, 0.1, 0.2, and 0.125 g (accurate to 0.0001 g) of the above-mentioned adsorption materials were weighed and added into the centrifuge tubes (with 3 parallel samples set for each addition amount). The centrifuge tubes were oscillated at a constant temperature of 25 °C and a speed of 220 rpm/min in a constant-temperature water-bath shaker. After 24-h adsorption equilibrium, the centrifuge tubes were taken out and placed in a centrifuge at 4000 r/min for 5 min. A certain amount of the supernatant was taken and filtered under reduced pressure using a suction-filtration pump through a 0.45-μm water-based filter membrane. Inductively-coupled plasma mass spectrometry was used to determine the Cd^2+^ concentration of each solution sample. Then, the adsorption amount and removal rate of Cd^2+^ by each biochar under different addition amount conditions were calculated.

### 4.5. Data Analysis

#### 4.5.1. Calculation of Adsorption Amount and Removal Efficiency

The adsorption effect of rice husk biochar on pollutants is represented by the adsorption amount (Q_e_) and removal efficiency (E), with the calculation formulas shown in Equations (1) and (2), respectively:

(1) $$Q_{e}=\frac{(c_{0}-c_{e})V}{m}$$

(2) $$E=(\frac{c_{0}-c_{e}}{c_{0}})\times 100\%$$

where Q_e_ is the adsorption amount of the adsorbent at adsorption equilibrium, in mg·g^−1^; c_e_ is the pollutant concentration in the adsorbent at adsorption equilibrium, in mg·L^−1^; c_0_ is the initial pollutant concentration, in mg·L^−1^; V is the volume of the pollutant, in mL; m is the mass of the added biochar, in g; E is the removal efficiency, in percent.

#### 4.5.2. Adsorption Kinetic Models

Pseudo-first order and pseudo-second order kinetic models were used to analyze the adsorption process and calculate the adsorption rate, as shown in Equations (3) and (4), respectively:

(3) $$\mathrm{Pseudo}\text{-}\mathrm{first}\;\mathrm{order}\;\mathrm{kinetic}\;\mathrm{model}:\;ln(Q_{e}-Q_{t})=lnQ_{e}-k^{1}t$$

(4) $$\mathrm{Pseudo}\text{-}\sec\mathrm{ond}\;\mathrm{order}\;\mathrm{kinetic}\;\mathrm{model}:\;\frac{t}{Q_{t}}=\frac{1}{k_{2}Q_{e}^{2}}+\frac{t}{Q_{e}}$$

where Q_e_ is the adsorption amount of the adsorbent at adsorption equilibrium, in mg·g^−1^; Q_t_ is the adsorption amount of the adsorbent at time t, in mg·g^−1^; k^1^ is the pseudo-first order adsorption rate constant, in min^−1^; k_2_ is the pseudo-second order adsorption rate constant, in g·mg^−1^·min^−1^.

#### 4.5.3. Intraparticle Diffusion Model

Based on the results of the adsorption kinetic experiments, the intraparticle diffusion equation is fitted, as shown in Equation (5):

(5) $$Q_{t}=k_{ip}t^{0.5}+c$$

In the equation, Q_t_ is the adsorption amount of the adsorbent at time t, in mg·g^−1^; k_ip_ is the intraparticle diffusion adsorption rate constant, in g·mg^−1^·min^−0.5^; c is the constant, in mg·g^−1^, indicating that the biochar boundary layer decreases with the increase in the biochar surface heterogeneity and hydrophilic groups, the larger the c value indicates the greater the influence of the boundary layer on adsorption. If intraparticle diffusion occurs during the adsorption process, then a plot of Q_t_ against t^0.5^ will be a straight line; if the line passes through the origin, then intraparticle diffusion is the only rate-limiting factor.

#### 4.5.4. Adsorption Isotherm Models

The adsorption isotherm models selected for this study were the Langmuir and Freundlich models, as shown in Equations (6) and (7), respectively.

(6) $$\mathrm{Langmuir}\;\mathrm{equation}:\;Q_{e}=\frac{Q_{\max}K_{L}c_{e}}{1+K_{L}c_{e}}$$

(7) $$\mathrm{Freundlich}\;\mathrm{equation}:\;Q_{e}=K_{F}{c_{e}}^{\frac{1}{n}}$$

In the equations: Q_max_ is the maximum adsorption amount for a monolayer on the adsorbent, in mg·g^−1^; c_e_ is the solution concentration at adsorption equilibrium, in mg·L^−1^; K_L_ is the Langmuir constant, in L·mg^−1^, indicating adsorption affinity, with K_L_ approaching 0 indicating irreversible adsorption; K_F_ is the Freundlich adsorption constant; $\frac{1}{n}$ is the parameter in the Freundlich model indicating adsorption intensity, with 0 < n < 1 being favorable for adsorption, n > 1 being unfavorable for adsorption, and n = 1 being a linear distribution.

Data processing was performed using Excel 2021, significant difference analysis was conducted using SPSS 17.0, and model fitting and plotting were performed using Origin 2022.

## 5. Conclusions

The functional group varieties within the three modified biochars were largely in accord with those of the unmodified biochar. A350 and C350 exhibited a conspicuous augmentation in pore quantity, whereas B350 presented a more even surface with predominantly filled pores. A350 experienced a reduction in polarity, accompanied by a diminished number of oxygen-containing functional groups, thereby leading to a weakened hydrophilic nature. In contrast, B350 and C350 displayed an increase in polarity, along with a greater number of oxygen-containing functional groups, resulting in enhanced hydrophilicity. The Zeta potential analysis revealed that A350 bore a positive charge across all pH values, intimating that the complexation reaction might be the principal factor underlying the adsorption of Cd^2+^ by A350. C350 holds a negative charge at any pH, accompanied by a more pronounced electrostatic repulsion between particles, which is conducive to the adsorption of metal cations. XRD analysis demonstrated that A350 presented a feeble diffraction peak of chitosan crystals; C350 showed a crystal diffraction peak of ammonia; and A350, B350, and C350 all possessed crystal diffraction peaks of selenium elements, signifying the successful incorporation of selenium onto the biochar.

The adsorption kinetics of B350 and C350 with respect to Cd^2+^ were more congruent with the pseudo-second order model, while the adsorption kinetics of A350 for Cd^2+^ adhered to both the pseudo-first order and pseudo-second order models. The isothermal adsorption characteristics of B350 and C350 were more in harmony with the Langmuir model, whereas the isothermal adsorption characteristics of A350 were more in line with the Freundlich model. The fitting outcomes of the intraparticle diffusion model were all three-stage, with monolayer chemical adsorption predominating. A350 exhibited the highest maximum adsorption capacity (57.845 mg/g), exceeding that of C350 (39.666 mg/g) and B350 (25.799 mg/g). All three modification approaches, to a certain extent, enhanced the Cd^2+^ adsorption capacity of the rice husk biochar.

As the pH ascended, the removal rate and adsorption quantity for CK350, A350, B350, and C350 all experienced an increment. With an increase in the dosage, the removal rate for CK350, A350, B350, and C350 rose, while the unit adsorption amount declined. By modulating the solution pH and dosage (for instance, from neutral to slightly alkaline, with a dosage of 0.01 g), the Cd^2+^ adsorption efficacy of the modified rice husk biochar can be adjusted.

## Figures and Tables

**Table 1:** Elemental content of rice husk biochar and selenium-modified rice husk biochar.

Sample	Mass Percentage/%						
	C	N	O	Si	P	Se	Cd
CK350	77.75	2.26	15.72	2.51	0.95	0.08	0.21
A350	77.59	2.81	13.05	2.02	0.89	4.58	0.15
B350	69.91	4.70	17.75	2.04	0.88	3.64	0.08
C350	62.42	3.48	28.53	1.59	1.16	2.75	0.07

**Table 2:** Fitting parameters of adsorption kinetic models for rice husk biochar and selenium-modified rice husk biochar.

Adsorption Kinetics	Parameter	CK350	A350	B350	C350
Pseudo-first order kinetic model	q_e_/(mg·g^−1^)	9.422	41.535	22.015	36.771
	k_1_	0.451	1.387	0.802	1.083
	R^2^	0.897	0.923	0.895	0.889
Pseudo-secondary kinetic model	q_e_/(mg·g^−1^)	10.379	44.472	24.233	36.211
	k_2_	0.060	1.894	0.088	0.075
	R^2^	0.956	0.966	0.951	0.923

**Table 3:** Fitting parameters of the intraparticle diffusion model for rice husk biochar and selenium-modified rice husk biochar.

Item	Parameter	CK350	A350	B350	C350
The first stage	k_ip_/(mg·g^−1^·min^0.5^)	6.363	39.718	19.594	42.75
	c/(mg·g^−1^)	1.897	6.848	5.251	15.038
	R^2^	1	1	1	1
The second stage	k_ip_/(mg·g^−1^·min^0.5^)	4.147	6.267	6.39	7.857
	c/(mg·g^−1^)	0.933	26.998	6.564	18.432
	R^2^	0.998	0.964	0.973	0.976
The third stage	k_ip_/(mg·g^−1^·min^0.5^)	0.121	0.287	0.341	0.048
	c/(mg·g^−1^)	8.896	41.578	21.236	37.481
	R^2^	0.969	0.720	0.762	0.877

**Table 4:** Fitting parameters of the isothermal adsorption models for rice husk biochar and selenium-modified rice husk biochar.

Adsorption Isotherms	Parameter	CK350	A350	B350	C350
Langmuir model	q_max_/(mg·g^−1^)	12.256	57.845	25.799	39.666
	K_1_(L·mg^−1^)	0.049	0.040	0.417	0.951
	R^2^	0.970	0.868	0.994	0.989
Freundlich model	K_F_	1.962	7.504	11.322	19.538
	1/n	0.363	0.402	0.198	0.183
	R^2^	0.861	0.953	0.791	0.836

## Data Availability

The original contributions presented in this study are included in the article.
